# Detection technology and clinical applications of serum viral products of hepatitis B virus infection

**DOI:** 10.3389/fcimb.2024.1402001

**Published:** 2024-07-05

**Authors:** Ying Liu, Di Wu, Kui Zhang, Rongrong Ren, Yuxuan Liu, Shuya Zhang, Xuanyu Zhang, Jilin Cheng, Liping Chen, Jun Huang

**Affiliations:** ^1^ School of Life Sciences, Zhengzhou University, Zhengzhou, Henan, China; ^2^ State Key Laboratory of Resource Insects, College of Sericulture, Textile and Biomass Sciences, Southwest University, Chongqing, China; ^3^ Department of Clinical Laboratory, Zhejiang Hospital, Hangzhou, Zhejiang, China; ^4^ Department of Gastroenterology, Shanghai Public Health Clinical Center, Fudan University, Shanghai, China; ^5^ Department of Gastroenterology, Shanghai Geriatric Medical Center, Shanghai, China

**Keywords:** HBV, serum viral products, detection technology, diagnosis, treatment

## Abstract

Viral hepatitis, caused by its etiology, hepatitis virus, is a public health problem globally. Among all infections caused by hepatitis-associated viruses, hepatitis B virus (HBV) infection remains the most serious medical concern. HBV infection particularly affects people in East Asia and Africa, the Mediterranean region, and Eastern Europe, with a prevalence rate of > 2%. Currently, approximately 1 billion people worldwide are infected with HBV, and nearly 30% of them experience chronic infection. Chronic HBV infection can lead to chronic hepatitis B (CHB), liver cirrhosis, and hepatocellular carcinoma (HCC), resulting in the related death of approximately 1 million people annually. Although preventative vaccines and antiviral therapies are currently available, there is no cure for this infection. Clinical testing is not only the gateway for diagnosis of HBV infection, but also crucial for judging the timing of medication, evaluating the effect of antiviral therapy, and predicting the risk of relapse after drug withdrawal in the whole follow-up management of hepatitis B infected persons. With advances in detection technology, it is now possible to measure various viral components in the blood to assess the clinical status of HBV infection. Serum viral products of HBV infection, such as HBV DNA, HBV RNA, hepatitis B surface antigen, hepatitis B e-antigen, and hepatitis B core-related antigen, are non-invasive indicators that are critical for the rapid diagnosis and management of related diseases. Improving the sensitivity of monitoring of these products is essential, and the development of corresponding detection technologies is pivotal in achieving this goal. This review aims to offer valuable insights into CHB infection and references for its effective treatment. We provide a comprehensive and systematic overview of classical and novel methods for detecting HBV serum viral products and discusses their clinical applications, along with the latest research progress in this field.

## 1 Introduction

**Figure 1:**
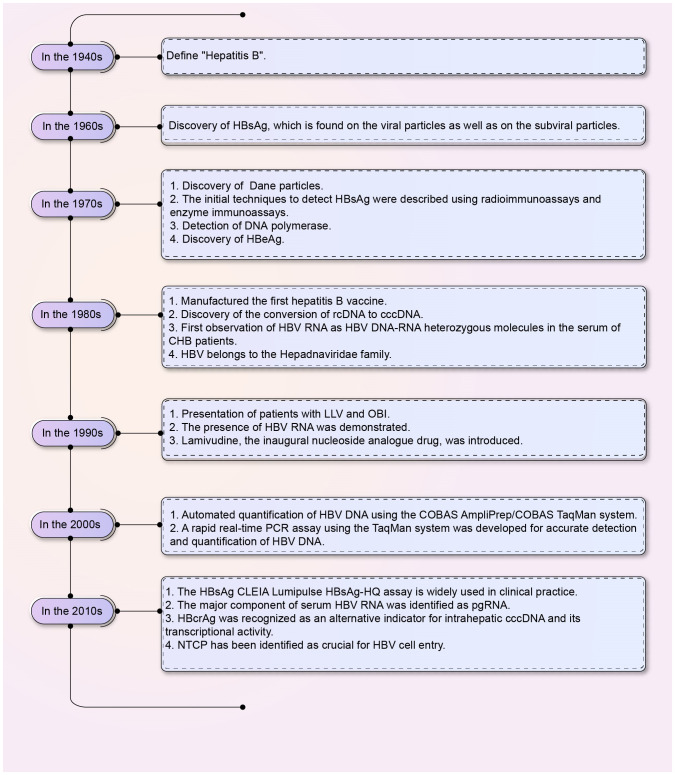
The discovery of HBV serum viral products. The chart shows the discovery of HBV serum viral products from the 1940s to the 2010s. HBV, Hepatitis B virus; HBsAg, HBV surface antigen; cccDNA, Covalently closed circular DNA; rcDNA, Relaxed circular DNA; HBeAg, Hepatitis B e-antigen; CHB, Chronic hepatitis B; CLEIA, Chemiluminescent enzyme immunoassay; pgRNA, Pre-genomic RNA; LLV, Low-level viremia; OBI, Occult hepatitis B virus infection; HBcrAg, Hepatitis B core-related antigen; NTCP, Sodium taurocholate co-transporting polypeptides.

Hepatitis B virus (HBV) is a 3.2 kb partially double-stranded DNA virus that belongs to the *Hepadnaviridae* family (Barker et al., 1975; Li et al., 2015). The discovery of the “Australia antigen,” now known as the hepatitis B virus surface antigen (HBsAg), by Dr. Baruch Blumberg in the 1960s paved the way for the diagnosis, prevention, and treatment of HBV infection (Blumberg, 1964; Blumberg et al., 1965; Li et al., 2020). The discovery history of serum viral products of HBV was shown in **Figure 1**. The European Association for the Study of the Liver (EASL) spliced chronic HBV infection into the following categories: hepatitis B e-antigen (HBeAg)-positive chronic infection (formerly known as the immune tolerance period), HBeAg-positive chronic hepatitis (formerly known as the HBeAg-positive immune activity period or immune clearance period), HBeAg-negative chronic infection (formerly known as the inactive carrier phase or low replication phase), and HBeAg-negative chronic hepatitis (formerly known as the HBeAg-negative immune active phase or reactivation phase) based on the evaluation of HBV-related liver disease indicators (**Table 1**) (European Association for the Study of the Liver, 2017; Chinese Society of Hepatology, 2022a; Zhuang, 2022). However, approximately 40% of patients cannot be categorized under the stages mentioned above (Chinese Society of Hepatology, 2022a); consequently, a new category of infection, referred to as the “uncertain period” of chronic HBV infection, has been defined. Furthermore, the risk of chronic hepatitis B (CHB) progression in patients during the “uncertain period” remains high (Chinese Society of Hepatology, 2022a).

**Table 1:** Natural history of chronic HBV infection (Chinese Society of Hepatology, 2022a; Jung & Nguyen, 2023; Zhuang, 2022).

Variable	Chronic infection		Chronic hepatitis	
	HBeAg positive	HBeAg negative	HBeAg positive	HBeAg negative
HBsAg	High	Low	High/Medium	Medium
HBeAg	Positive	Negative	Positive	Negative
HBV DNA	>10^7^ IU/ml	<2000 IU/ml	10^4^-10^7^ IU/ml	>2000 IU/ml
ALT	Normal	Normal	Increase	Increase continuously or intermittently
Hepatic lesions	None/Mild	None	Moderate/Severe	Moderate/Severe

**Figure 2:**
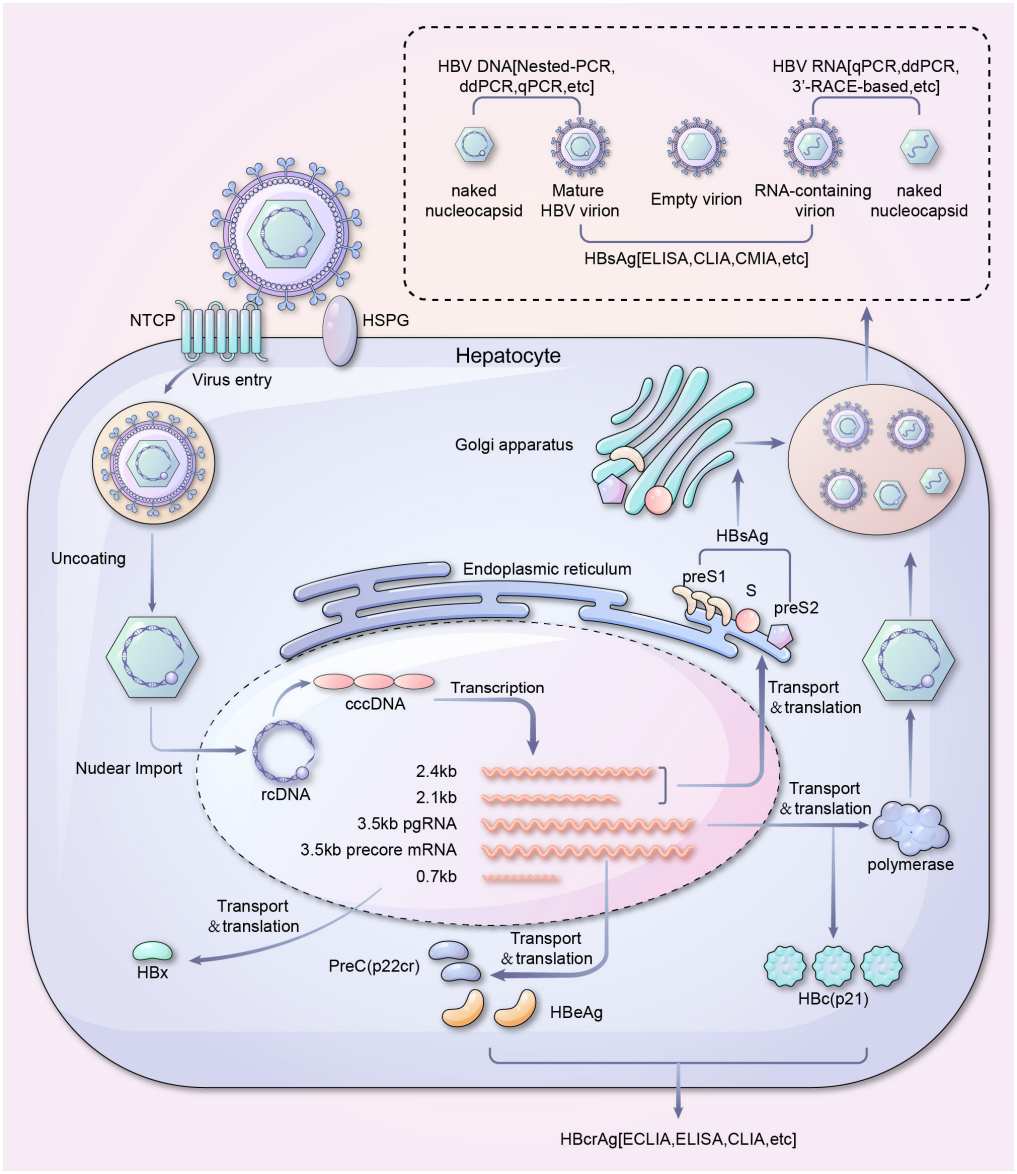
Life cycle and serum viral products detection of HBV. A schematic overview of serum viral products in HBV life cycle and their corresponding detection methods. It should be noted that covalently closed circular DNA (cccDNA) is the template for all the HBV mRNAs, while integrated HBV DNA can transcribe X and S/preS2/preS1 RNAs only. ddPCR, Droplet digital PCR; qPCR, Quantitative polymerase chain reaction; HBsAg, HBV surface antigen; ELISA, Enzyme-linked immunosorbent assay; CLIA, Chemiluminescence immunoassay; CMIA, Chemiluminescent microparticle immunoassay; NTCP, sodium taurocholate co-transporting polypeptide; HSPG, Heparan sulfate proteoglycan; ECLIA, Electrochemiluminescence immunoassay; cccDNA, Covalently closed circular DNA; rcDNA, Relaxed circular DNA; HBeAg, Hepatitis B e-antigen.

Over the past 30 years, there has been extensive research on the life cycle of HBV. The virus binds to liver-specific receptors such as sodium taurocholate co-transporting polypeptides and heparan sulfate proteoglycans, resulting in endocytosis and the release of HBV core particles (Yan et al., 2012; Sureau and Salisse, 2013; Wu and Chang, 2015). These particles are transported to the nucleus, where the HBV genome is released and gets converted from relaxed circular double-stranded DNA (rcDNA) to covalently closed circular DNA (cccDNA), which is highly stable and contributes to the persistent HBV infection and low cure rates (Gerlich and Robinson, 1980; Molnar-Kimber et al., 1983; Tuttleman et al., 1986; Tsukuda and Watashi, 2020). HBV cccDNA interacts with host transcription factors to produce pre-genomic RNA (pgRNA), HBV X mRNA, pre-core mRNA, and preS/S mRNA (Su et al., 1989a; Su et al., 1989b; Nassal, 2015). These HBV RNAs encode viral proteins, including surface (HBs), precore or e (HBe), and core (HBc) antigen, polymerase, and X (HBx) proteins (Moon et al., 2024). The HBV mRNA is primarily transported to the cytoplasm and translated (Wieland et al., 2000; McCoullough et al., 2024). pgRNA is translated into core proteins and viral polymerase in the cytosol. The pgRNA and the viral polymerase are encapsidated. The pgRNA is further reverse-transcribed to rcDNA by the HBV polymerase within the nucelocapsids (core particles) (Bruss, 2007; SChadler and Hildt, 2009; Imam et al., 2018). Mature core particles re-migrate to the nuclear pore complex or are enveloped by viral surface proteins and secreted by multivesicular bodies (MVB) (Babst, 2005; Watanabe et al., 2007; Pan et al., 2023). Early assessment of histological damage and residual levels of *de novo* infection caused by viral activity during the above HBV life cycle is crucial for preventing hepatitis recurrence and liver disease progression and ultimately achieving a functional cure(Maintain negative Hepatitis B surface antigen, undetectable HBV DNA, and normal liver biochemical indicators)against CHB infection (Author Group of Expert, 2022). The life cycle of HBV and its corresponding detection methods were summarized and shown in **Figure 2**.

## 2 Advancements in the methods for detecting serum viral products

In clinical practice, based on the nature of specific viral products, valuable information for evaluating the phase of HBV infection can be obtained by quantifying serum viral products. **Table 2** listed the detection methods for HBV serum viral products. Classical detection methods include the following: quantitative polymerase chain reaction (qPCR), enzyme-linked immunosorbent assay (ELISA), radioimmunoassay (RIA), gold immunochromatography assay (GICA), and time-resolved fluoroimmunoassay (TRFIA) (Hu et al., 2012; Maity et al., 2012; Irshad et al., 2016; Jiang et al., 2020; Dera et al., 2023). Novel stable and accurate detection technologies, including chemiluminescence immunoassay (CLIA), chemiluminescent microparticle immunoassay (CMIA), microparticle enzyme immunoassay (MEIA), and automatic chemiluminescent enzyme immunoassay (CLEIA), have also emerged (Ollier et al., 2008; Khadem-Ansari et al., 2014; Liu et al., 2014; Ghosh et al., 2015; Amini et al., 2017; Inoue et al., 2021).

**Table 2:** Clinical application of detection methods in HBV infection.

Detection methods	Advantages	Clinical application	Disadvantages
qPCR	Fast, specific and sensitive (Heid et al., 1996; Hu et al., 2023a).	Direct detection of the HBV-DNA in serum (Wikstrom et al., 2000; Ji et al., 2020).	High requirements for personnel and working environment (Lequin, 2005; Hu et al., 2023a).
RIA	High sensitivity and specificity (Kroes et al., 1991; Kimura et al., 2002).	HBsAg, HBsAb, HBeAg, HBeAb, HBcAb can be detected (Utiger et al., 1962; Yin et al., 2012; Dong et al., 2013; Huang et al., 2017).	RIA can easily lead to radioactive contamination and unstable reagents (Alves et al., 2017; Kim et al., 2021).
ELISA	Simple operation, high sensitivity, strong specificity and good reproducibility (Engvall and Perlmann, 1971; Lequin, 2005).	HBsAg and other HBV serum markers in the blood is usually qualitative detected (Engvall et al., 1971; Lequin, 2005).	Temporary reading and having lots of influencing factors (Engvall and Perlmann, 1971; Lequin, 2005).
GICA	Simple and quick operation, easy preservation, providing the possibility of detection of HBsAg in peripheral blood (Liubavina et al., 2007; Wang et al., 2014).	GICA was only suitable for the preliminary screening of HBsAg positive individuals (Wang et al., 2014; Dogbe and Arthur, 2015; Kalita et al., 2022; Ti et al., 2022).	Low sensitivity (Liubavina et al., 2007; Wang et al., 2014).
TRFIA	Wider linear range, low background and high specificity (Halonen et al., 1983; Hu et al., 2012).	Due to the high sensitivity of the TRFIA method, the very low amount of HBsAg in the patient’s body can be detected (Deguchi et al., 2004; Zhao et al., 2022).	Complicated operation (Deguchi et al., 2004).
CLIA	The label has long validity period, simple operation, easy to realize full automation, and reduce human operation error, strong specificity (Yang et al., 2019).	Detection of HBV serum markers (including HBsAg, anti-HBs, HBeAg, anti-HBe, anti-HBc, etc.) (Loglio et al., 2020; Li et al., 2022).	Reagent cost is high, instrument cost is expensive (Khadem-Ansari et al., 2014; Loglio et al., 2020; Li et al., 2022).
MEIA	High sensitivity, strong specificity, good repeatability and easy operation (Duverlie et al., 1988; Zhao et al., 2022; Chen et al., 2024).	Accepted reference method for the quantitative determination of HBV serological markers (Zhao et al., 2022; Chen et al., 2024).	High cost (Zhao et al., 2022; Chen et al., 2024).
CMIA	Automatic operation, high throughput, fast (Deguchi et al., 2004; Chen et al., 2024).	Widely used in the quantitative detection of serological markers of HBV infection (Deguchi et al., 2004; Hadziyannis et al., 2008).	High cost (Deguchi et al., 2004; Chen et al., 2024).

Detection methods for HBV serum viral products can be divided into molecular biological detection and immunological detection methods (Rong et al., 2021).qPCR is a robust molecular biological detection method that involves the addition of a fluorescent gene to the PCR reaction system (Heid et al., 1996). This allows for real-time monitoring of the entire PCR process using fluorescence signal accumulation, thereby facilitating the accurate detection of an unknown template using a standard curve (Caliendo et al., 2011; Siegel et al., 2023). Compared to clinical detection, molecular biological detection and other assays, qPCR-based detection has a relatively low degree of automation and requires high levels of expertise from operators and careful management of experimental conditions (Paraskevis et al., 2010; Caliendo et al., 2011; Irshad et al., 2016; Liu et al., 2019). Using TaqMan probes, HBV RNA in serum samples can be quantified using qPCR (Ji et al., 2020). However, several influencing factors such as homologous and heterologous DNA backgrounds and oligonucleotide hybridization specificity, which may cause quantitative bias in qPCR reactions, need to be considered during qPCR operation. Accordingly, to ensure the accuracy and reliability of the results, it is crucial to optimize the experimental conditions during the operation (Wikstrom et al., 2000; Tay et al., 2001).

RIA is an immunoassay that has been used in clinical practice since the 1960s (Yalow and Berson, 1960; Utiger et al., 1962). It is based on the principle that the labeled and non-labeled antigens (to be tested) bind competitively to a limited number of specific antibodies; the greater the radioactivity of the Ag-Ab complex, the more the labeled antigen binds and the lower is the concentration of non-labeled antigen (to be tested) (von der Waart et al., 1978). This detection method has the characteristic of good cross-reflection. However, clinical trials have shown that RIA can easily lead to radioactivity-related contamination and cannot quickly complete detection operations.

ELISA has become ubiquitous in medical laboratories, *in vitro* diagnostic product manufacturing industries, regulatory agencies, and external quality assessment and capability verification organizations (Engvall and Perlmann, 1971; Engvall et al., 1971; Lequin, 2005; Maity et al., 2012). It is an immunological detection method commonly used to detect the presence of HBV in patients. ELISA, while valuable and cost-effective for virus detection, is predominantly marketed for qualitative analysis at present. Additionally, it is prone to problems such as false negatives due to hook effects (Lequin, 2005; Maity et al., 2012; Hu et al., 2023a). GICA has the advantages of simple operation, rapidity, and easy preservation (Jiang et al., 2007). However, its sensitivity is relatively low, which may lead to missed detection. TRFIA is a novel ultramicron immunoassay method that integrates the advantages of ELISA and RIA (Halonen et al., 1983). It can quantitatively detect HBV serum viral products with high specificity and sensitivity and samples with low level of replication, thus avoiding missed detection. It should be noted that TRFIA has a significant disadvantage, which is complicated operation (Fu et al., 2020).

CLIA is a widely used technology in clinical medicine for detecting trace amounts of antigens and antibodies (Velan and Halmann, 1978). It is a labeled immunoassay that combines luminescence analysis with immune system reactions (Khadem-Ansari et al., 2014; Shen et al., 2019). CLIA emerged after ELISA and RIA. Due to its simple operation, convenient marking, high stability and sensitivity, high speed, and low environmental impact, it has been well-received by medical examiners and doctors in clinical practice. Electrochemiluminescence immunoassay (ECLIA), a susceptible detection technology that combines electrochemical luminescence with immunoassays, is dependent on chemiluminescence reactions and is a subtype of CLIA. Its significant stability and sensitivity make it ideal for detecting trace substances (Kovac et al., 2020). However, ambiguous results obtained from ECLIA-based detection need to be further confirmed using additional detection methods. CLEIA, a type of CLIA, is a detection method wherein a photon signal is generated via the interaction of an enzyme labeled on an antigen or antibody with a luminescent substrate. Generally, horseradish peroxidase and alkaline phosphatase (ALP) are the two most common enzyme markers used in CLEIA. In practice, its use is limited by the extensive sample quality requirements and high reagent costs. MEIA is an immunoassay technique that uses microparticle endofactors to form a complex with the substance to be tested (Duverlie et al., 1988). The complex then reacts with an ALP conjugate compound to produce a fluorescent product. It has the advantages of high sensitivity, specificity, reproducibility, and simplicity of operation. CMIA is a technique that involves two methods, competitive and double antibody sandwich (Amini et al., 2017). The small solid-phase magnetic particles used in this technique have a diameter of only 1.0 μm. This small size increases the coating surface area, amount of adsorption of the antigen or antibody, and reaction speed and reduces the probability of pollution and cross-contamination (Chen et al., 2005; Li et al., 2022). In CMIA, the antigen or antibody is labeled with ALP, which undergoes luminescence reactions by reacting with its substrate, dioxane phosphate (Loglio et al., 2020). CMIA includes a variety of serum immunoassays with high sensitivity (as low as 0.1 ng/mL) for detecting HBsAg with good repeatability and specificity (Hadziyannis et al., 2008; Motyka et al., 2022). However, the cost of related equipment is high (Motyka et al., 2022).

## 3 Routine serum viral products of HBV and their detection methods

A critical aspect of managing CHB infection involves monitoring the process of HBV replication (Burns and Thompson, 2014). Serological and histological products are typically detected during the diagnosis of HBV infection. Throughout the infection, cccDNA accumulates in the nucleus, persists as a stable inclusion, and serves as a template for the transcription of viral genes (Ding et al., 2023). Several studies have suggested that HBV cccDNA has a relatively short half-life (Lu et al., 2017; Gao et al., 2022); however, its persistence remains a significant challenge in eradicating HBV infection. Another major challenge in HBV cccDNA research is the absence of an efficient method that can directly detect cccDNA in liver biopsy tissues with high sensitivity, significant specificity, and accurate quantification (Tu et al., 2021). To address this issue and facilitate the study of cccDNA, various new methods, including PCR-based methods and *in situ* hybridization, have recently been applied (Li et al., 2017). PCR-based methods encompass conventional qPCR, competitive qPCR, semi-nested and nested qPCR, and droplet-digital PCR (ddPCR), among others. These methods offer advantages of being simpler, faster, more accurate, cost-effective, sensitive, and capable of higher throughput. *In situ* hybridization is capable of distinguishing and locating different DNA and RNA. Proteins can be identified in conjunction with the method by utilizing immunohistochemistry (IHC) or immunofluorescence. Serological detection is currently considered an alternative approach; it is non-invasive, easy to operate, and cost-effective. Accordingly, finding an ideal serological biomarker that can reflect the presence of HBV cccDNA and its transcriptional activity is a crucial clinical requirement.

The detection and study of HBV serum viral products have significant value in diagnosing and treating HBV infection and play a crucial role in promoting the development of anti-viral therapies. Currently, common serological products that are used to diagnose HBV infection include HBsAg, anti-HBs, HBeAg, anti-HBe, anti-HBc, and HBV DNA. Among these, HBsAg is a widely used viral product for diagnosing hepatitis B and is considered superior to other viral products in terms of indicating clinical cure after treatment with pegylated interferon and nucleos(t)ide analogs (Chuaypen et al., 2018; Lim et al., 2021; Wang et al., 2023). HBsAg is an antigen that is found on the surface of HBV; it comprises small, medium, and large HBV proteins. In addition, HBsAg is self-assembled in non-infectious subviral particles (SVPs) which are secreted in a large excess compared to the viral particles (Boulon et al., 2020). This antigen plays a crucial role in initiating the infection process of HBV by facilitating the attachment of the virus to the host cell membrane (Marquardt et al., 1987; Pontisso et al., 1989; Lee and Ahn, 2011). HBsAg is the earliest serological product to appear in patients with acute hepatitis B infection. It is also frequently detected in chronic disease carriers, indicating ongoing viral transcriptional activity (Blumberg et al., 1967; Kao, 2008; de Almeida Ponde, 2022). CLEIA, CMIA, CLIA, or standard ELISA is generally performed to detect HBsAg (Takeda et al., 2013; Kim, 2017). Chronic HBV infection is defined as a persistent infection lasting more than six months, during which HBsAg can be detected (Chinese Society of Hepatology, 2022b). Patients with low levels of HBsAg may still experience active replication of the virus and associated liver injury and may also be capable of spreading the infection (Wu et al., 2021; Moini and Fung, 2022). As diagnostic technologies and treatment for HBV continue to advance, researchers are increasingly focusing on individuals with low levels of HBsAg (Wu et al., 2022). It has been shown that a combination of HBsAg quantification and the expression of certain T-cell markers could be a potential predictor of HBsAg clearance in patients with chronic HBV infection within 12 months (Wang et al., 2023). CMIA-based quantitative analysis of HBsAg demonstrates high sensitivity in detecting not only free HBsAg protein but also antigen-antibody complexes and mutant forms of HBsAg.

Hepatitis B e-antigen (HBeAg) is a soluble component of the hepatitis B core antigen. The presence of HBeAg indicates the risk of active replication of HBV and transmission of the infection (Liaw et al., 2010; Cornberg et al., 2017). Clinical laboratories detect HBeAg using procedures such as ELISA, ECLIA, or CLIA. Research studies focusing on these methods have demonstrated that HBeAg quantification is a valuable tool in predicting the antiviral efficacy of HBV reactivation. Additionally, HBeAg levels are higher in patients experiencing HBV reactivation than in those with acute infection and HBeAg-positive chronic infection (Piermatteo et al., 2021). HBeAg seroconversion is a crucial goal encompassing antiviral treatment for patients with CHB. When a patient undergoes HBeAg seroconversion, it indicates that HBV has entered the low-level replication stage, which further indicates a reduction in the likelihood of progression to cirrhosis and liver cancer as well as the risk of infectivity (Hadziyannis and Laras, 2018). Several factors can influence the seroconversion of HBeAg. Numerous cytokines/chemokines and other indicators, including IL-37, IP-10, IL-21, and CLEC18, are correlated with HBeAg seroconversion in patients with CHB who are being treated with nucleos(t)ide analogues (NAs) (Ma et al., 2012; Guo et al., 2016; Zhao et al., 2021). Studies conducted on mice demonstrated that in Kupffer cells, HBeAg inhibited the transcription of NLRP3 and pro-interleukin 1β by reducing the phosphorylation level of NF-κB. Additionally, it inhibited caspase-1 activation and IL-1β maturation by blocking the production of reactive oxygen species (Yu et al., 2017).

High levels of HBV DNA in the serum of a patient indicate active replication of the virus in the liver, which is a critical factor in the progression of liver disease. Recent research suggests that even persistently low levels of HBV DNA can contribute to CHB progression (Sun et al., 2020). The detection of HBV DNA in the serum of a patient is considered the most reliable method for determining hepatitis B viremia; additionally, HBV DNA in the serum is the most reliable product of active viral replication (Robinson et al., 1974; Raimondo et al., 2019). The level of HBV DNA in the serum of a patient is closely associated with the risk of developing liver fibrosis and hepatocellular carcinoma (HCC) in patients with CHB (Kim et al., 2022). Over the past decade, significant advancements have been made in the detection methods used to assess serum HBV DNA levels, resulting in increased sensitivity of detection. Currently, in addition to nested PCR and ddPCR, qPCR is the most widely used method for detecting secreted HBV DNA (Wang et al., 2014; Saitta et al., 2022; Zhang and Tu, 2022). Liver samples in every instance must undergo proper processing to prevent cross-contamination. Moreover, inclusion of suitable negative controls is essential to validate the assay’s specificity.

## 4 Novel serum viral products of HBV and their detection methods

Recently, there has been a growing trend toward using novel serological viral nucleic acid products, such as serum HBV RNA, for monitoring the clinical status of HBV patients. Previous studies indicated that HBV RNA in serum is pgRNA, which is encapsulated in HBV-like virus particles that can be secreted extracellularly. This suggests that HBV may have an alternative form of virion, in which the nucleic acid is composed of RNA rather than DNA (Wang et al., 2016). HBV RNA has been identified as an indicator of intrahepatic transcriptional activity of cccDNA and was found to be associated with liver histological changes in patients with CHB who have been treated with nucleoside (acid) analogs (Wang et al., 2016; Wang et al., 2017; Jiang et al., 2020). Serum HBV RNA levels have emerged as a useful alternative for assessing the transcriptional activity of cccDNA. Numerous studies have shown that although HBV DNA is below the detection limit, or HBsAg has seroconverted, HBV RNA still exists. Therefore, compared to HBV DNA and other indicators, HBV RNA has higher clinical value in evaluating the efficacy of anti-HBV therapy, selecting the timing for discontinuation of treatment, and predicting the risk of recurrence after cessation of treatment.

A case-control study involving 104 patients receiving entecavir (ETV) treatment revealed that after adjusting for various risk factors such as age, sex, presence or absence of cirrhosis, and duration of antiviral therapy, the level of HBV RNA during treatment was associated with an increased risk of developing HCC within the next two years (Carey et al., 2020; Fan et al., 2020; Dahari et al., 2021; Lok et al., 2022). To date, relatively few studies have been published on the association between HBV RNA levels and the risk of developing HCC. It has been reported that HBV RNA serves as a predictive product not only for HBsAg response during early antiviral therapy but also for the risk of HBsAg reversal after discontinuing the treatment (Wang et al., 2018). Intrahepatic HBV RNA levels approaching those of inactive carriers are also considered a useful viral endpoint for discontinuation of NA therapy. Additionally, HBV RNA should be used as an indicator for discontinuation of testing; this is also included in several guideline consensus (Wang et al., 2017; Berg and Petersen, 2018). At 24 weeks, HBV RNA levels declined more rapidly in patients who received HBsAg serologic conversion than in those who did not (Mak et al., 2022). Several different techniques, including rapid amplification RT-qPCR based on cDNA terminal (RACE), qPCR, and ddPCR, are available for quantifying RNA levels (Limothai et al., 2020; Lok et al., 2022; Yu et al., 2022; Hu et al., 2023b). However, standardization of HBV RNA detection methods is essential. Various experimental methods exist for quantifying intrahepatic and serum HBV RNA, all relying on quantitative RT-qPCR assays. However, consensus remains elusive regarding a singular technical or commercial assay for HBV RNA detection (Hu et al., 2023b).

Hepatitis B core-related antigen (HBcrAg) includes several proteins, including HBV core antigen, HBeAg, and pre-core/core protein (p22cr), and has a molecular weight of 22 kDa (Kimura et al., 2005; Hong et al., 2021; Adraneda et al., 2023). Its quantitative measurement is of great significance for guiding the clinical management of chronic HBV infection (Lee and Ahn, 2011; Ye et al., 2021; Adraneda et al., 2023). HBcrAg has emerged as a novel product of CHB infection and is correlated with the responses to current antiviral therapies for HBeAg-positive CHB; this product should be considered in addition to secreted HBV RNA when evaluating new antiviral therapies that directly or indirectly target hepatic cccDNA, with the goal of achieving functional cure (Chuaypen et al., 2016; Wong et al., 2017; Kramvis et al., 2022). The use of HBcrAg detection methods in patients with undetectable HBV DNA and HBsAg is anticipated to become a beneficial prognostic factor for determining the long-term prognosis of patients with CHB infection (Honda et al., 2016; Watanabe et al., 2021; Sonneveld et al., 2022). The predicted performance of HBcrAg may vary depending on the clinical endpoint being considered for CHB infection (Tseng et al., 2023). The clinically anticipated performance of HBcrAg is inconsistent, and there is a poor correlation between HBsAg loss and antiviral treatment (Wong et al., 2023). Therefore, HBcrAg-related results should be interpreted carefully in clinical practice (Tseng et al., 2022; Adraneda et al., 2023). CLEIA is a method primarily used for detecting HBcrAg in patient serum. CLEIA detects a combination of HBcAg, HBeAg (both free and in the HBeAg–HBe antibody complex), and precore proteins in blood, validated for dried blood spot (Wang et al., 2007).

## 5 Summary

Over the past decade, there has been significant progress in evaluating the progression of non-invasive liver disease in CHB patients. Although existing antiviral drugs for treating hepatitis B can effectively control the progression of such diseases, they can rarely eliminate the virus or achieve functional cure (Fanning et al., 2019; Chinese Society of Hepatology, 2022a; Watanabe et al., 2022). Additionally, cccDNA may still exist in the nuclei of the livers of patients, increasing their likelihood of HBV reactivation and developing HCC (Wang et al., 2014; Moro et al., 2018; World Health, 2019). The levels of novel HBV products have been demonstrated to correlate with the regression and prognosis of CHB disease. The levels of novel serological viral products, including HBV RNA, HBcrAg, and cccDNA, along with their transcriptional activity, can serve as exploratory endpoints in new drug research (Testoni et al., 2023).. However, the detection of these innovative serum viral products faces methodological challenges. Novel serum viral products can be compared or combined with routine serum viral products (such as HBV DNA, HBeAg, and HBsAg) to assess disease progression clinically. However, the detection should be conducted in centralized labs using thoroughly validated standardized reagents and platforms, accompanied by comprehensive detection protocols (Kuhns et al., 2021). Novel serum viral products aid in analyzing the mechanism of new therapeutic drugs. Anticipated technological advancements and the progress in ultrasensitive assays might potentially redefine the meaning of “functional cure” or even “partial functional cure” in the foreseeable future (Kramvis et al., 2022; Lok et al., 2022).

Overall, the detection of novel serum viral products allows for assessing antiviral effectiveness and predicting the relapse risk after drug withdrawal. This assists clinicians in providing better treatments for patients with CHB. Here, we systematically describe the traditional serum products of HBV (HBsAg, HBeAg, and HBV DNA) and the emerging serum viral products (HBV RNA and HBcrAg) and discuss their detection methods and applications. The principles of these detection methods vary widely, and each has its own unique advantages and disadvantages. CLIA is a non-radioactive immunoassay method that has rapidly advanced in the past 30 years. Owing to the high sensitivity of chemiluminescence and strong specificity of immunoassays, it has attracted wide attention recently. CLIA is widely used in clinical diagnosis and biochemical analysis to detect various tumor markers, cytokines (Yang et al., 2019), and hormones. Its sensitivity and specificity have been improved at both the qualitative to quantitative levels, its procedure has been fully automated, and the detection time has been remarkable reduced. The ECLIA method has ideal clinical application value, with advantages of high detection rates and a wide detection range. The reagents utilized in ECLIA remain long-term stability without the risk of toxicity or contamination. Currently, advanced chemiluminescence systems are being manufactured by Abbott, Siemens, Roche, Beckman, and other international brands as well as domestic brands, such as Mindray and Avron.

## 6 Prospect

Several reports indicate that 27.8% to 59.5% of chronic HBV-infected patients are in “uncertain period” (Chinese Society of Hepatology, 2022b). Patients with chronic HBV infection in the “uncertain period” are at higher risk of progression of liver fibrosis, cirrhosis, and HCC than those without “uncertain period.” The value of noninvasive liver fibrosis assessments (e.g., hepatic transient elastography) in evaluating progression in “uncertain” chronic HBV-infected patients is debatable. Certainly, new serologic assays are being investigated, and better results are expected for clinical guidance. Among patients with CHB infection undergoing antiviral therapy, although potent low-resistance oral antiviral therapy results in potent suppression of HBV replication, low-level viremia (LLV) persists in some patients. Additionally, more LLV is present in some patients in the “uncertain period”. LLV is a term used to describe the detection of HBV DNA in the serum of a patient at levels ranging from 20 to 2000 IU/ML after 48 weeks of antiviral treatment (European Association for the Study of the Liver, 2017; Terrault et al., 2018). According to the guidelines of the American Association for Liver Research and the EASL, potent antiviral drugs such as tenofovir alafenamide fumarate, tenofovir, and ETV are recommended for the management of LLV, after excluding the issues related to compliance and detection errors (Lee et al., 2022). “Uncertain period” LLV refers to the “gray zone” between inactive carriers and HBeAg-negative CHB in patients with first-treatment chronic HBV infection who, after 1 year of follow-up, exhibit a pattern of HBV DNA and ALT that differs from the patterns of the four traditional stages of chronic HBV infection, with levels of either HBV DNA or ALT intermediate between those of inactive carriers and those of HBeAg-negative CHB (Yan and Sun, 2023). Highly sensitive detection methods such as ECLIA and CLIA for HBsAg or HBeAg are needed for patients in this period.

High sensitivity detection of HBsAg and HBV DNA significantly influences the selection and adjustment of antiviral therapy, aiding in predicting efficacy, guiding cessation, and enhancing transfusion safety. High sensitivity method also identifies OBI and shortens the window period for detecting acute HBV infection. Patients with OBI are negative for serum HBsAg, while HBV DNA is present in the liver with detectable/undetectable levels in the serum (Chinese Society of Hepatology, 2022b). Im YR et al. found that the prevalence of OBI reflects the prevalence of Hepatitis B: 0.98% in high prevalence areas, 0.12% in moderately prevalent areas as well as 0.06% in low prevalence areas (Im et al., 2022). OBI has been associated with advanced chronic liver disease, especially HCC, and patients with OBI can transmit HBV (Chen et al., 2019; Saitta et al., 2022; Wu et al., 2024). Combining novel serum viral products with routine serum viral products improves the sensitivity and specificity of identifying OBI and predicting the presence of OBI in the liver. Clinically, HBsAg and HBV DNA are two important serum viral products necessary for HBV diagnosis based on the infection’s natural history. Many treated patients primarily use standard sensitivity detection, considering high sensitivity as supplementary. There is a significant cost difference between the two methods; the high sensitivity detection method has a higher clinical value than the ordinary sensitivity detection; however, considering the economic burden of patients, the ordinary sensitivity detection can be applied to most of the patients.

HBV has high genetic diversity due to different genotypes and even intergenotypic recombinants (Bollyky et al., 1996; Ghosh et al., 2013; Liao et al., 2017; Liu et al., 2018; Feng et al., 2020). Genotypes may influence disease progression, drug resistance, response to antiviral therapy and prognosis. Intergenotype recombination is an important mechanism of virus evolution. Naturally occurring deletions/insertions have been found in the HBV core promoter, which may affect the production of core antigens (Peng et al., 2015). The HBV S gene tends to accumulate immune escape mutations, which may interfere with the use of immunological methods for clinical detection (Ding et al., 2014; Liu et al., 2024). Sun et al. showed that mutations in the pre-S region of the HBV genome may be associated with the development of OBI. The pre-S mutants of genotype B located in the pre-S2/S promoter significantly reduced the production of HBsAg by influencing the promoter activity, thus promoting the occurrence of OBI (Sun et al., 2022). Further, Jiang et al. found that the high frequency mutation of S protein transmembrane domain may be related to the occurrence of OBI (Jiang et al., 2022). It has been shown that mutations in precore, basal core promoter and preS in HBeAg-negative patients are associated with quantitative HBsAg serum levels and HBV DNA levels (Li et al., 2010; Kuhnhenn et al., 2018). Deletion/insertion mutations in the whole genome of HBV are prevalent in HBeAg-positive CHB patients prior to antiviral therapy, and the higher the detection rate of these mutations, the better the response to lamivudine and adefovir dipivoxil combination therapy (Hao et al., 2015). However, more studies are needed to reveal the impact of HBV genetic diversity and genotype on the production and detection of HBV serum markers.

## Author contributions

YiL: Conceptualization, Data curation, Formal analysis, Investigation, Methodology, Resources, Software, Validation, Visualization, Writing – original draft. DW: Conceptualization, Data curation, Formal analysis, Investigation, Methodology, Resources, Software, Validation, Visualization, Writing – original draft. KZ: Conceptualization, Investigation, Methodology, Validation, Writing – review & editing. RR: Conceptualization, Formal analysis, Methodology, Resources, Writing – review & editing. YuL: Conceptualization, Data curation, Investigation, Methodology, Resources, Visualization, Writing – original draft. SZ: Conceptualization, Investigation, Methodology, Software, Writing – original draft. XZ: Conceptualization, Data curation, Investigation, Methodology, Software, Validation, Writing – original draft. JC: Conceptualization, Investigation, Methodology, Validation, Visualization, Writing – review & editing. LC: Conceptualization, Investigation, Methodology, Software, Supervision, Validation, Writing – review & editing. JH: Conceptualization, Funding acquisition, Project administration, Supervision, Writing – review & editing.

## Glossary

**Table:**

HBV	Hepatitis B virus
HBsAg	Hepatitis B virus surface antigen
EASL	European Association for the Study of the Liver
HBeAg	Hepatitis B e-antigen
CHB	Chronic hepatitis B
rcDNA	Relaxed circular DNA
cccDNA	Covalently closed circular DNA
pgRNA	Pre-genomic RNA
qPCR	Quantitative polymerase chain reaction
ELISA	Enzyme-linked immunosorbent assay
RIA	Radioimmunoassay
GICA	Gold immunochromatography assay
CLIA	Chemiluminescence immunoassay
MEIA	Microparticle enzyme immunoassay
CLEIA	Chemiluminescent enzyme immunoassay
ALP	Alkaline phosphatase
IHC	Immunohistochemistry
ETV	Entecavir
HBcrAg	Hepatitis B core-related antigen
LLV	Low-level viremia
OBI	Occult hepatitis B virus infection
ddPCR	Droplet digital PCR
ECLIA	Electrochemiluminescence immunoassay
HCC	Hepatocellular carcinoma
